# P10 A new paradigm for treating refractory and recurrent infectious vaginal discharge

**DOI:** 10.1093/jacamr/dlaf118.017

**Published:** 2025-07-14

**Authors:** Paul F Long

**Affiliations:** Institute of Pharmaceutical Science, King’s College London, London, UK

## Abstract

**Background:**

The most common causes of infectious vaginitis are bacterial vaginosis (BV) and vulvovaginal candidiasis (VVC), with BV being of polymicrobial dysbiosis origin and VVC caused by various *Candida* species. BV is typically treated with prescription antibiotics, either in tablet form or incorporated into creams, gels and pessaries, or over-the-counter (OTC) acid-containing personal healthcare products. Treatment for VVC usually consists of an OTC single antifungal containing tablet or antifungal containing creams and gels. However, there is high likelihood that BV and VVC will reoccur when using these treatment approaches. Not only is antimicrobial resistance a growing concern, but antibiotics and antifungals are indiscriminate in their action, which causes a further imbalance in the vaginal microbiome and a never-ending cycle of symptoms that often pivot between BV and VVC (both diseases can even occur simultaneously in some women). In the absence of new, more potent antimicrobial agents to eradicate drug-resistant pathogenic vaginal microbiota, we herein describe an alternative approach that uses microbiome remodelling for the treatment of refractory and recurrent infectious vaginitis.

**Methods:**

A mucoadhesive hydrogel was prepared using a hot and cold hydration process. Vaginal swabs freshly collected from women with clinically and laboratory diagnosed BV and/or VVC were exposed to the gel. Microbiology culture dependent and independent approaches were used to assess if the gel promoted commensal *Lactobacillus* growth and dominance but, importantly, not to stimulate growth of BV- or VVC-causing organisms.

**Results:**

Culture dependent and independent methods demonstrated the gel supported regrowth of lactobacilli to numbers equivalent to that expected in a healthy vagina. Culture independent methods showed that not only do numbers of lactobacilli increase but there is also a shift in species diversity towards types found in women less likely to suffer repeated episodes of BV or VVC. Culture dependent experiments also demonstrated that the gel supported recovery of statistically significant lactobacilli in greater numbers from vaginal swabs of women with confirmed BV than the market leading brand of OTC acid containing vaginal gel.

**Conclusions:**

A novel mucoadhesive hydrogel intended to be eventually marketed as an OTC medical device called ‘DUO Balance’ by BiotaLife Healthcare Ltd envisages an innovative natural approach to treat both BV and VVC by restoring microbiome balance in the vagina without any need of antibiotics, antifungals or acid components. This approach has been successfully tested *in-vitro* and patented, and it prevents any possibility to drive antifungal or antibiotic resistance and other unpleasant adverse reactions (e.g. irritation due to the introduction of acid) or frequent relapses, unlike other currently available therapeutic options.

